# Integrated Phytoremediation and Microbial Mitigation Strategies for Sustainable Restoration of Toxic Metal-Contaminated Soils

**DOI:** 10.3390/biology15191732

**Published:** 2026-10-01

**Authors:** Inès Hammami, Mohamed Abdelaal, Ibtisam M. Ababutain, Sumayah I. Alsanie, Ghadah H. Al Hawas, Azzah I. Alghamdi, Nadiyah M. Alqazlan, Ruba A. Alghamdi, Fajir M. Alsubaie, Joaher Y. Alnabbat

**Affiliations:** 1 Department of Biology, College of Science, Imam Abdulrahman Bin Faisal University, P.O. Box 1982, Dammam 31441, Saudi Arabia; ihammami@iau.edu.sa (I.H.); iababutain@iau.edu.sa (I.M.A.); galhawas@iau.edu.sa (G.H.A.H.); azalghamdi@iau.edu.sa (A.I.A.); nmalqazlan@iau.edu.sa (N.M.A.); rubaghamdi6@gmail.com (R.A.A.); fajir.056@gmail.com (F.M.A.); joaheran@gmail.com (J.Y.A.); 2 Basic and Applied Scientific Research Center, Imam Abdulrahman Bin Faisal University, P.O. Box 1982, Dammam 31441, Saudi Arabia

**Keywords:** microbiome, omics, sustainable soil restoration, CRISPR, nanotechnology

## Abstract

Toxic metal contamination of soil remains a critical global challenge due to the persistence, bioaccumulation, and irreversible toxicity of these metals. Cadmium (Cd), lead (Pb), mercury (Hg), arsenic (As), chromium (Cr) and nickel (Ni) are the most common toxic metals that threaten food security, ecosystem stability, and human health. While conventional physicochemical remediation technologies provide rapid pollutant removal, they are constrained by high economic costs, secondary pollution risks, and long-term soil degradation. Biological approaches, particularly phytoremediation and microbial mitigation, offer environmentally compatible alternatives; however, their field-scale performance is often limited by low biomass production, restricted metal bioavailability, microbial instability, and heterogeneous soil conditions. This review critically synthesizes recent advances that move beyond standalone biological strategies toward integrated, multi-scale remediation frameworks. We evaluate the mechanistic basis and performance of nano-assisted phytoremediation, biochar-mediated stabilization, omics-guided plant–microbe optimization, and clustered regularly interspaced short palindromic repeats/CRISPR-associated protein systems (CRISPR/Cas) for enhancing metal transport, chelator biosynthesis, antioxidant defense, and microbial detoxification pathways. Emerging evidence indicates that nano-enabled amendments and biochar composites can reduce bioavailable Cd fractions by up to 75%, while omics-driven microbial consortia and targeted genome editing improve tolerance and accumulation efficiency in key crops such as rice and sorghum. Importantly, we assess translational barriers including nanoparticle ecotoxicity, off-target genome effects, regulatory constraints, biomass disposal, and life-cycle sustainability that currently limit large-scale deployment. By integrating mechanistic insights with field validation data, risk assessment considerations, and technology readiness evaluation, this review proposes a conceptual roadmap for scalable, eco-efficient soil restoration. The convergence of nanotechnology, systems biology, microbiome engineering, and precision genome editing represents a transformative pathway for next-generation remediation strategies capable of restoring contaminated soil while safeguarding environmental and public health.

## Simple Summary

Toxic metal contamination of soils poses a global threat because metals such as Cd, Pb, Hg, As, Cr, and Ni threaten ecosystems, food security, and human health. Although conventional remediation methods are effective, they may cause secondary pollution or soil degradation. Biological approaches such as phytoremediation and microbial mitigation are more sustainable but perform inconsistently under field conditions. This review evaluates integrated strategies combining plants and microorganisms with nano-enabled materials, biochar, omics-guided optimization, and CRISPR/Cas engineering. These approaches can enhance metal extraction or immobilization, plant tolerance, detoxification, and soil recovery. The review examines ecological, regulatory, economic, and biosafety constraints and proposes a site-specific framework for sustainable remediation that achieves durable reductions in contaminant exposure and ecological risk.

## 1. Introduction

Toxic metals (TMs) are metals of environmental and toxicological concern. Soil contamination by toxic metals represents one of the most persistent and globally pervasive environmental challenges of the 21st century. In contrast to many biodegradable organic contaminants, toxic metals such as cadmium (Cd), lead (Pb), mercury (Hg), arsenic (As), chromium (Cr), and nickel (Ni) cannot be mineralized into harmless end products, and are capable of long-term accumulation in soils [1,2]. Their environmental behavior is instead governed by changes in oxidation state, speciation, complexation, precipitation, adsorption, and biological sequestration. Their persistence facilitates transfer across the soil–plant–animal continuum, resulting in biomagnification within food webs and chronic exposure risks to human populations [2,3]. Accelerated industrialization, mining activities, fossil fuel combustion, wastewater irrigation, agrochemical overuse, and urban expansion have significantly intensified metal inputs into agricultural and peri-urban soils worldwide [4,5]. Recent global assessments estimate that approximately 14–17% of cropland exceeds safe metal thresholds, posing potential health risks to nearly 1.4 billion people [3].

Conventional physicochemical remediation technologies including excavation, soil washing, vitrification, and chemical stabilization can achieve rapid contaminant removal or immobilization [6,7,8]. However, these approaches are frequently constrained by high operational costs, energy intensity, disruption of soil structure, and the generation of secondary pollutants [4,6,9]. Chemical amendments such as phosphates, lime, and chelating agents may reduce bioavailable fractions but can alter soil physicochemical properties and raise concerns regarding long-term stability and leaching risks [10,11,12,13]. Consequently, increasing attention has shifted toward sustainable, nature-based remediation strategies capable of restoring soil functionality while minimizing environmental trade-offs.

Biological approaches, particularly phytoremediation and microbe-mediated detoxification, have emerged as promising alternatives due to their cost-effectiveness, ecological compatibility, and potential for in situ application [1,14,15]. Phytoremediation exploits plant physiological mechanisms such as phytoextraction, phytostabilization, rhizofiltration, and phytovolatilization to remove, immobilize, or transform metal contaminants [16,17,18]. In parallel, microorganisms contribute through biosorption, bioaccumulation, biomineralization, and redox transformations that modify metal speciation and bioavailability [19,20,21]. Increasing evidence highlights that synergistic plant–microbe interactions significantly enhance remediation efficiency by improving plant biomass, nutrient acquisition, and tolerance under metal stress [21,22,23].

Despite these advances, several critical bottlenecks continue to limit field-scale implementation. Hyperaccumulator plants often exhibit low biomass and slow growth rates, restricting total metal removal capacity [17,18]. Metal bioavailability in aged or heterogeneous soils is frequently insufficient to sustain efficient phytoextraction [9,24]. Introduced microbial inoculants may fail to establish stable populations under fluctuating environmental conditions, reducing long-term effectiveness [20,25]. Furthermore, remediation processes are typically time-intensive and lack standardized performance metrics for field validation and scalability assessment [26,27]. These constraints highlight the gap between promising laboratory findings and practical deployment in complex soil systems.

Over the past decade, several emerging technologies have been proposed to overcome these limitations. Nanotechnology-assisted phytoremediation may enhance metal solubility, transport, and uptake through engineered nanoparticles and nano-chelators with high surface reactivity [28,29,30]. Biochar and composite amendments can improve soil structure, water retention, and microbial habitat while stabilizing labile metal fractions [31,32]. Omics-based approaches, including metagenomics, transcriptomics, proteomics, and metabolomics, provide system-level insights into plant–microbe–metal interactions and enable the identification of key transporters, resistance genes, and metabolic pathways for targeted optimization [33,34]. Additionally, CRISPR/Cas-mediated genome editing has opened new possibilities for engineering plants and microorganisms with enhanced metal tolerance, transporter regulation, and detoxification capacity [35].

Despite rapid progress in phytoremediation, microbial remediation, nanotechnology, and genome engineering, existing reviews have largely evaluated these approaches in isolation [14,15,20,27,29,30,35]. A critical unresolved challenge is therefore how to integrate these technologies into site-specific remediation systems while reconciling mechanistic compatibility, field robustness, ecological safety, regulatory feasibility, economic viability, and technology readiness. Addressing this translational gap requires an analytical framework that connects mechanistic evidence with strategy selection and operational deployment.

Accordingly, this review aims to: (i) synthesize the physiological, biochemical, and molecular mechanisms underlying plant- and microbe-mediated toxic metal remediation; (ii) critically evaluate the functional integration of phytoremediation and microbial mitigation, including potential synergistic and antagonistic interactions; (iii) assess the roles of nano-enabled amendments, biochar, multi-omics approaches, and CRISPR-based engineering in optimizing integrated remediation systems; and (iv) examine the ecological, regulatory, economic, and technology-readiness barriers governing their field-scale translation.

## 2. Conceptual Gap, Novelty and Analytical Framework

Rather than providing another descriptive catalog of individual remediation technologies, this review develops a decision-oriented, multilevel framework for translating mechanistic advances into site-specific remediation strategies. The framework integrates four interdependent decision layers: (i) site characterization based on contaminant identity, concentration, speciation, bioavailability, distribution, and soil properties; (ii) definition of the primary remediation endpoint, including extraction, stabilization, transformation, exposure reduction, or restoration of soil function; (iii) selection and assembly of functionally compatible plant genotypes, microbial inoculants or consortia, and soil amendments; and (iv) evaluation of field performance, ecological risk, economic and life-cycle feasibility, regulatory acceptability, and technology readiness.

The novelty of this review lies in explicitly linking mechanistic evidence to strategy selection, field deployability, and measurable remediation outcomes. This framework distinguishes laboratory efficacy from operational readiness and identifies the conditions under which integrated systems may provide advantages over standalone treatments. It also clarifies critical trade-offs, for example, between metal mobilization for phytoextraction and immobilization—that may otherwise increase phytotoxicity, contaminant leaching, food-chain transfer, or long-term ecological risk. By connecting biological mechanisms, site constraints, intended endpoints, and translational requirements, the framework provides a structured basis for designing integrated remediation systems that are not only effective under controlled conditions but also safe, scalable, and sustainable under field-relevant conditions.

## 3. Methodological Approach

This review adopted an integrative narrative approach to critically examine recent advances in phytoremediation, microbial-assisted remediation, and collective plant–microbe strategies for the sustainable restoration of soils contaminated with toxic metals. Emphasis was placed on the mechanisms regulating metal uptake, immobilization, transformation, and detoxification; plant–microbe interactions in the rhizosphere; the application of soil amendments and emerging technologies; and the environmental, economic, and practical limitations affecting field-scale implementation.

### 3.1. Literature Search and Selection

**Figure 1:**
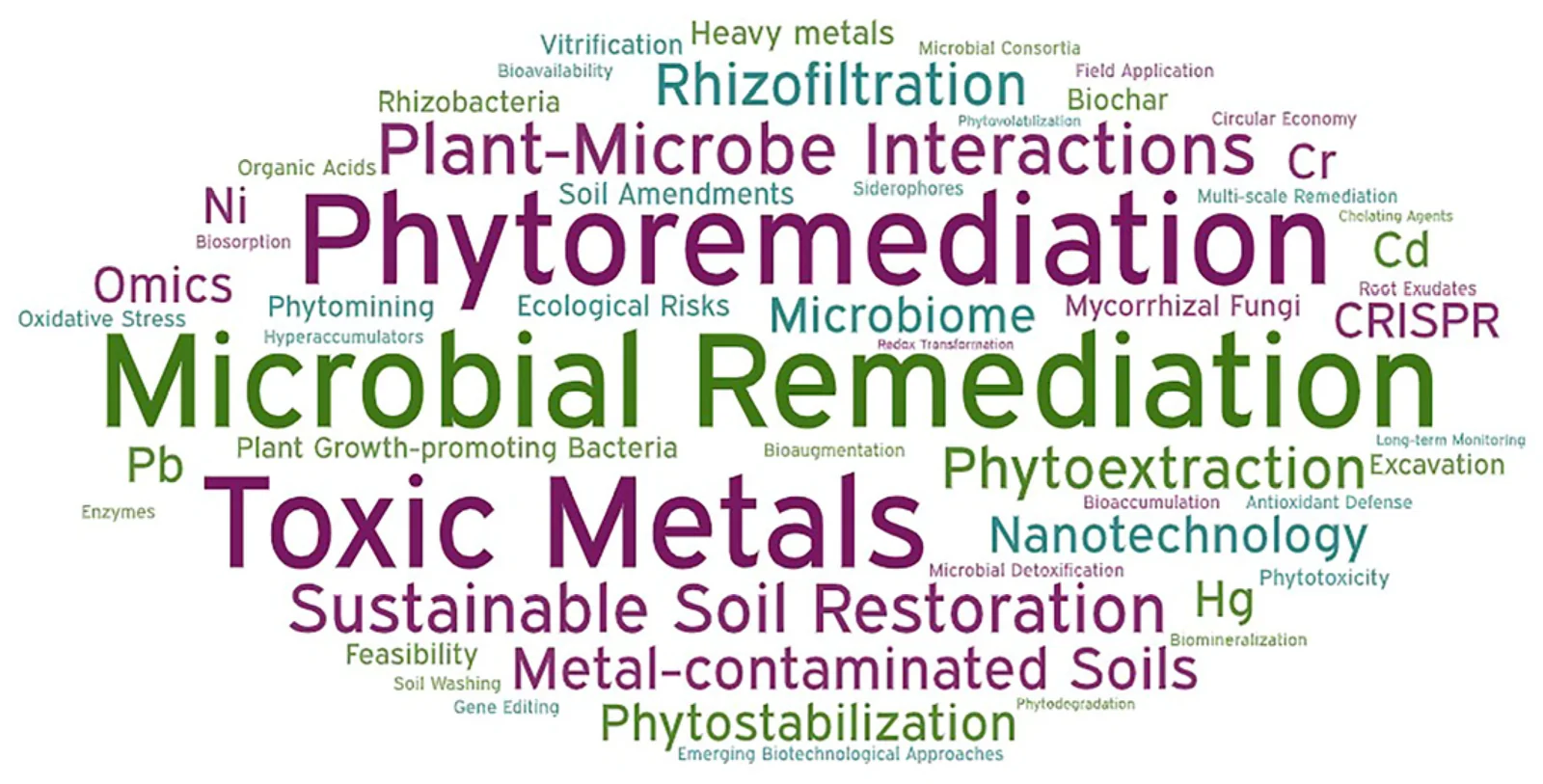
The major keywords and thematic concepts associated with integrated phytoremediation and microbial mitigation of toxic metal-contaminated soils.

The relevant peer-reviewed literature was identified through searches of Scopus, Web of Science (WOS) and Google Scholar. Searches were conducted using different keywords including phytoremediation, phytoextraction, phytostabilization, toxic metals, CRISPR, metal-contaminated soils, plant growth-promoting bacteria, microbial remediation, plant–microbe interactions, biochar, soil amendments, nanotechnology, phytomining, and sustainable soil restoration, etc. (Figure 1). Additional combinations were used to recognize studies addressing integrated remediation systems, field-scale applications, ecological risks, economic feasibility, and emerging biotechnological approaches.

Priority was given to peer-reviewed research articles and recent review papers published during (2015–2025), while earlier formative studies were included where necessary to provide mechanistic or historical context. The selected literature was critically evaluated and systematized according to major thematic areas, including the sources and behavior of toxic metals in soils; mechanisms of phytoremediation; microbial mechanisms involved in metal immobilization, transformation, and plant-growth promotion; synergistic plant–microbe interactions; amendment-assisted remediation; and emerging approaches such as nanotechnology, engineered microbial consortia, genome-editing technologies, and phytomining. The literature was further examined with respect to remediation efficiency, sustainability, ecological safety, scalability, treatment duration, biomass management, technology readiness, and economic feasibility.

### 3.2. Use of Generative Artificial Intelligence

Generative artificial intelligence was used solely to help in the preparation of conceptual figures. ChatGPT-5.3 (OpenAI, San Francisco, CA, USA; image generation model: GPT-image) was provided with author-developed prompts describing the scientific concepts, biological interactions, and remediation processes to be illustrated. The generated outputs were refined through iterative prompting and subsequently reviewed and edited by the authors. Scientific accuracy, terminology, and conceptual relationships were verified against the peer-reviewed literature, and unsupported or inaccurate elements were corrected or removed.

## 4. Soil Toxic-Metal Contamination: Sources, Types, and Global Status

Soil contamination by toxic metals is a globally pervasive environmental issue driven by both natural geochemical processes and intensified anthropogenic activities (Table 1). While lithogenic inputs from parent material weathering, volcanic emissions, and atmospheric deposition contribute to baseline metal concentrations, human activities now represent the dominant source of excessive accumulation in most agricultural and industrial regions [36,37]. Rapid industrialization, mining and smelting operations, fossil fuel combustion, sewage sludge application, wastewater irrigation, electronic waste disposal, and intensive agrochemical use have substantially increased soil metal burdens over recent decades [4,5].

### 4.1. Classification and Toxicological Characteristics of Toxic Metals

Toxic metals of major environmental concern include Pb, Cd, Hg, Cr, As, and Ni. These elements differ in biological function, redox behavior, mobility, and toxicity. Essential micronutrients such as Ni, and Cr (III) play roles in enzymatic catalysis and plant metabolism at trace concentrations but become phytotoxic when thresholds are exceeded [41] (Broadley et al., 2007). In contrast, non-essential and highly toxic metals including Pb, Cd, Hg, As, and Cr(VI) pose significant ecological and human health risks even at low concentrations due to their high bioavailability and ability to interfere with cellular processes [44]. Metal toxicity mechanisms typically involve disruption of protein structure, interference with nutrient uptake, displacement of essential cations, oxidative stress induction through reactive oxygen species (ROS) generation, DNA damage, and suppression of enzyme activity [39,42]. Speciation strongly influences toxicity; for example, Cr (VI) is highly soluble and carcinogenic, whereas Cr (III) is comparatively less mobile and less toxic [42]. Similarly, inorganic arsenic species (As^3+^ and As^5+^) differ in mobility and plant uptake behavior, influencing risk profiles across agroecosystems [45].

### 4.2. Anthropogenic Sources and Contamination Pathways

Anthropogenic inputs dominate contemporary soil metal enrichment. Major sources include mining and smelting where release of tailings, slag, and atmospheric particulates are enriched in Pb, Cd, and As [36], and industrial discharge from electroplating, tanning, textile production, battery manufacturing, and petrochemical industries contribute Cr, Ni, Pb, and Hg contamination [4,5]. Agricultural practices such as phosphate fertilizers often contain Cd impurities; pesticides and livestock manure may introduce As [5,24]. Wastewater and sewage sludge application long-term irrigation and sludge amendment increase Cd, Pb, and Hg levels in soils [37], urbanization and traffic emissions includes brake linings, tire wear, and fossil fuel combustion that contribute Pb deposition in peri-urban soils [5]. These sources frequently introduce metals in bioavailable or labile forms, accelerating accumulation within topsoil layers and increasing potential transfer into crops and groundwater systems [4,24].

### 4.3. Global Distribution and Contamination Trends

Toxic metal contamination patterns exhibit strong geographic variability depending on industrial history, land use practices, and geological background. Recent global assessments estimate that approximately 14–17% of agricultural soils exceed safe metal thresholds, affecting nearly 242 million hectares and exposing up to 1.4 billion people to elevated health risks [3]. Regional hotspots include East and South Asia where elevated Cd, Pb, and As concentrations associated with intensive agriculture and mining activities [3,45]. In Sub-Saharan Africa, Hg contamination is linked to artisanal and small-scale gold mining operations [36], in European industrial regions, Legacy Pb pollution in former smelting and mining zones [36], and Latin America (Andean region): high Cd, and As concentrations near mining districts [3]. Emerging research also highlights interactions between TMs and co-contaminants such as microplastics and persistent organic pollutants, which may alter metal mobility and bioavailability, complicating risk assessment and remediation strategies [46].

### 4.4. Soil Processes Governing Metal Mobility and Bioavailability

The environmental risk posed by TMs is governed not only by total concentration but also by chemical speciation, fractionation, and bioavailability. Key controlling factors include soil pH, redox potential, organic matter content, clay mineral composition, and cation exchange capacity [38]. Acidic conditions generally increase metal solubility, enhancing phytoavailability and leaching risk [38]. Conversely, alkaline conditions promote precipitation and adsorption onto mineral surfaces, reducing mobility. Organic matter may immobilize metals through complexation or enhance mobility via soluble organo-metal complexes depending on environmental conditions. Aging processes further influence metal behavior; over time, metals may become occluded within mineral matrices or incorporated into stable secondary phases, decreasing bioavailable fractions but complicating remediation efforts [24]. This dynamic interplay between chemical speciation and biological uptake underscores the need for remediation strategies that address both labile and stabilized fractions. Given their persistence, toxicity, and heterogeneous distribution, toxic metal contaminants pose long-term threats to agricultural productivity, soil biodiversity, and human health [3,47]. Importantly, total metal concentration alone is insufficient for risk evaluation; speciation analysis, bioaccessibility testing, and plant uptake studies are required to assess ecological and food-chain transfer risks [3,24]. Understanding the sources, chemical forms, and global distribution patterns of TMs provides the foundation for designing targeted phytoremediation and microbial mitigation strategies. As contamination intensifies under expanding industrial and agricultural pressures, scalable and sustainable remediation frameworks are increasingly essential to safeguard soil health and food security worldwide.

## 5. Ecological and Human Health Impacts

Toxic metal contamination exerts multi-scale impacts on soil ecosystems, plant productivity, and human health due to the persistence, bioaccumulation potential, and toxicity of metals such as Pb, Cd, Hg, Cr, As, and Ni (Table 1). Because these elements are non-degradable and remain in the soil for extended periods, their effects are chronic rather than transient, influencing ecological structure and function across contaminated regions.

### 5.1. Impacts on Soil Properties and Microbial Communities

Elevated concentrations of TMs alter soil physicochemical characteristics, disrupt nutrient cycling, and suppress biological activity [38]. As summarized in Table 1, contaminated soils across regions such as China, India, Poland, and the Iberian Peninsula show reduced microbial diversity, inhibited enzymatic activity, and impaired nitrogen fixation. TMs interfere with microbial metabolism by binding to functional groups of enzymes and structural proteins, resulting in decreased respiration rates and altered microbial community composition. Cadmium, lead, and mercury are particularly toxic to soil microorganisms, often reducing microbial biomass and suppressing key biogeochemical processes [48]. Such disruptions decrease soil fertility and resilience, undermining long-term agricultural sustainability. In regions affected by artisanal gold mining, such as Ghana and Nigeria (Table 1), Hg contamination has been associated with marked reductions in microbial diversity and ecological functionality. Similarly, in industrial zones of Saudi Arabia and the UAE, Pb, Ni, and Cr contamination correlates with suppressed soil enzyme activity and altered microbial structure (Table 1).

### 5.2. Phytotoxic Effects and Plant Growth Inhibition

In plants, TMs adversely affect plant physiological processes, leading to reduced germination, impaired root and shoot growth, chlorosis, and oxidative stress [41,42]. Table 1 highlights consistent plant-level responses across contaminated regions, including growth retardation, oxidative damage, impaired photosynthesis, and nutrient imbalance. Specific metals produce distinct effects: lead primarily accumulates in roots, limiting nutrient uptake; cadmium disrupts antioxidant defenses; mercury suppresses microbial activity; chromium (VI), due to its high solubility and reactivity, induces oxidative damage and cellular membrane disruption. Arsenic interferes with phosphate metabolism and energy production, particularly in paddy soils irrigated with contaminated groundwater, as reported in South Asia [45]; Ni inhibits chlorophyll synthesis [39,48]. In mining-intensive regions such as Peru, Chile, and the Democratic Republic of Congo (Table 1), the elevated Cd level is associated with chlorosis, reduced biomass accumulation, and inhibited root elongation. These physiological impairments translate directly into decreased crop yields and compromised food security [3].

### 5.3. Bioaccumulation and Trophic Transfer

A major ecological concern is the transfer of TMs from soil into plants and subsequently into animal and human food chains. Metals absorbed by crops can accumulate in edible tissues, increasing dietary exposure risk [39]. According to global assessments, significant portions of agricultural soils exceed safe thresholds for Cd and Pb, posing long-term risks to human populations [3]. Table 1 demonstrates that regions with elevated agricultural contamination such as China, India, and the Mississippi Basin (USA) report plant growth inhibition alongside documented risks of kidney dysfunction, neurotoxicity, and carcinogenicity in exposed populations. These patterns illustrate the direct linkage between soil contamination and public health outcomes.

### 5.4. Human Health Implications

Human exposure occurs through ingestion of contaminated crops, inhalation of dust particles, or consumption of polluted water. The health impacts vary depending on the specific metal and exposure duration. Lead (Pb) is associated with neurotoxicity, cognitive impairment, and cardiovascular disorders [38], Cd is linked to renal dysfunction and skeletal demineralization [39], Hg causes neurological deficits, particularly in mining-affected communities (Table 1), arsenic and Cr (VI) recognized carcinogens with long-term health implications [42,45], and Ni is associated with dermatitis and respiratory toxicity [39]. As indicated in Table 1, contamination hotspots consistently report neurological disorders, kidney damage, carcinogenic risks, and systemic toxicity. These health impacts are amplified in regions with prolonged exposure and limited environmental regulation.

### 5.5. Integrated Ecological Consequences

The ecological and human health effects of toxic metal contamination are interconnected. Suppressed microbial activity reduces nutrient cycling efficiency, which in turn affects plant growth and crop productivity. Impaired plants accumulate metals, facilitating trophic transfer and increasing dietary exposure risks. Over time, these cascading effects may result in land degradation, biodiversity loss, and socioeconomic burdens in contaminated regions [3]. The regional examples summarized in Table 1 underscore that toxic metal contamination is not only a localized environmental issue but a global ecological and public health concern. The persistence and cumulative nature of these impacts highlight the urgency of developing integrated, sustainable remediation strategies capable of restoring soil function while minimizing food-chain and human health risks.

## 6. Conventional Remediation Approaches and Their Limitations

**Figure 2:**
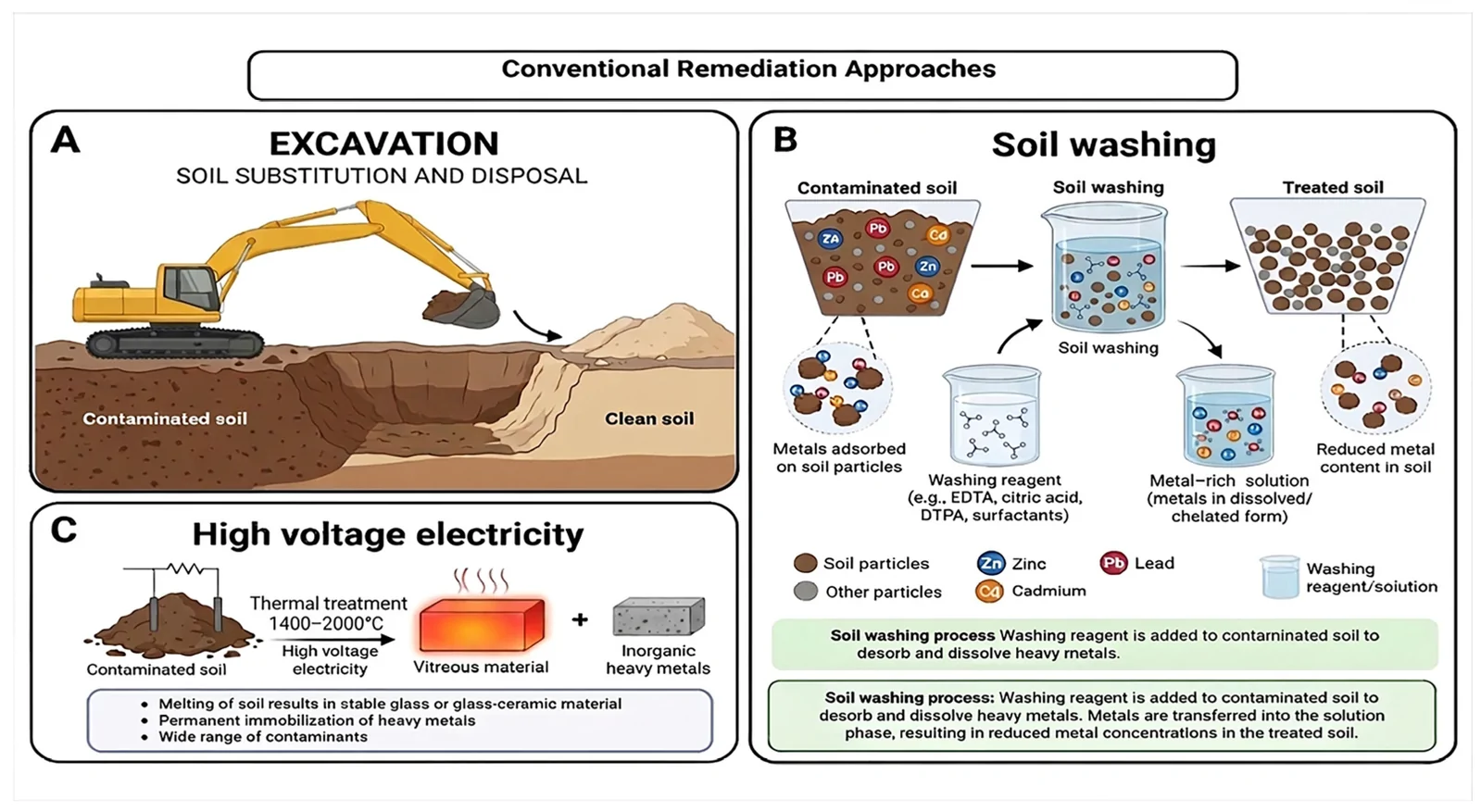
Conventional remediation approaches, (**A**) excavation, (**B**) soil washing, and (**C**) vitrification [4,6]. The figure was generated with the assistance of ChatGPT image generation and subsequently modified using BioRender.

Conventional remediation strategies for toxic metal-contaminated soils primarily rely on physical and physicochemical methods designed to remove, isolate, or immobilize contaminants. These approaches are generally characterized by rapid contaminant reduction and immediate site stabilization; however, they are often associated with high economic, environmental, and logistical costs [4,6]. As illustrated in Figure 2, the most widely applied conventional techniques include excavation, soil washing, and vitrification.

### 6.1. Excavation and Landfill Disposal

Excavation involves the physical removal of contaminated soil followed by transportation to landfills or secure disposal facilities (Figure 2A). This method provides immediate risk reduction and is frequently applied in heavily polluted industrial or mining sites [7]. Because contaminated material is physically removed, excavation can achieve near-complete elimination of localized contamination. Despite its effectiveness, excavation presents several critical limitations. The method is highly cost-intensive due to transportation, landfill fees, and site restoration requirements [4]. It disrupts soil structure, destroys native microbial communities, and eliminates beneficial soil biota, thereby compromising long-term soil functionality. Additionally, excavation merely transfers contamination from one location to another rather than achieving true detoxification. Dust dispersion during removal and potential secondary contamination during transport further complicate implementation. Consequently, while excavation may be suitable for small-scale or highly concentrated contamination hotspots, it is rarely feasible for large agricultural areas due to economic and ecological constraints.

### 6.2. Soil Washing

Soil washing is a physicochemical technique that separates contaminated soil fractions using aqueous solutions containing chelating agents, surfactants, acids, or bases to solubilize TMs [8]. Common chelating agents include ethylenediaminetetraacetic acid (EDTA), diethylenetriaminepentaacetic acid (DTPA), citric acid, and oxalic acid, which form stable complexes with metal ions and enhance desorption from soil particles [10,11]. This method can significantly reduce total metal concentrations and is particularly effective in sandy or coarse-textured soils where contaminants are loosely bound [8]. However, soil washing has several major drawbacks: generation of secondary wastewater requiring further treatment, loss of essential soil nutrients, alteration of soil physicochemical properties, and high operational and chemical costs. Moreover, chelating agents such as EDTA may increase metal mobility beyond the treatment zone, raising concerns about groundwater contamination [10]. These limitations restrict large-scale agricultural applications and necessitate careful post-treatment soil rehabilitation.

### 6.3. Vitrification

Vitrification is considered one of the most technologically advanced physical remediation methods. It involves heating contaminated soil to extremely high temperatures (approximately 1400–2000 °C), transforming it into a stable, glass-like matrix that immobilizes TMs within crystalline structures [49]. The process effectively reduces metal leachability and provides long-term stabilization. Despite its technical efficiency, vitrification is highly energy-intensive and expensive [6]. The requirement for specialized equipment and emission control systems further increases operational complexity. In addition, potential release of hazardous gases during high-temperature processing necessitates stringent environmental monitoring. These factors significantly limit their practical application to highly contaminated industrial sites rather than widespread agricultural lands.

### 6.4. Chemical Stabilization and Immobilization

Chemical remediation methods aim to reduce metal bioavailability through stabilization, precipitation, adsorption, or redox transformation [50]. Phosphate amendments, for example, promote the formation of insoluble pyromorphite, effectively immobilizing lead [12,13]. Lime applications increase soil pH, facilitating hydroxide and carbonate precipitation of metals such as Cd, and Ni [51]. Biochar–manganese oxide composites and zeolite amendments further enhance immobilization by providing high surface area and ion-exchange capacity [31,52]. While chemical stabilization reduces bioavailable fractions and ecological risk, it does not remove metals from the soil matrix. Long-term stability depends on soil conditions, and environmental changes such as pH fluctuation may re-mobilize previously stabilized metals [24]. Additionally, excessive chemical amendments may alter the soil structure, disrupt microbial communities, and reduce fertility over time [9].

### 6.5. Critical Limitations of Conventional Approaches

Although conventional remediation techniques provide rapid and measurable reductions in contaminant levels, they are constrained by several overarching limitations including high economic costs where equipment, reagents, energy consumption, and waste disposal significantly increase implementation expenses [4], secondary environmental impacts through generation of wastewater, emissions, or displaced contaminated materials may create additional environmental burdens. In addition, soil structure disruption where physical removal or aggressive chemical treatment damages soil aggregation and microbial ecosystems. Moreover, limited scalability of most conventional methods is impractical for large agricultural areas due to cost and logistical complexity. Finally, the lack of ecological restoration that focuses on contaminant removal or immobilization rather than restoring biological function and soil health. As a result, while conventional remediation remains essential for heavily polluted industrial sites, it is often unsuitable for sustainable, large-scale agricultural soil restoration. The ecological and economic limitations of these methods have driven increasing interest in biologically based strategies such as phytoremediation and microbial mitigation, which aim not only to reduce metal concentrations but also to rehabilitate soil functionality.

## 7. Chemical Remediation Methods

**Figure 3:**
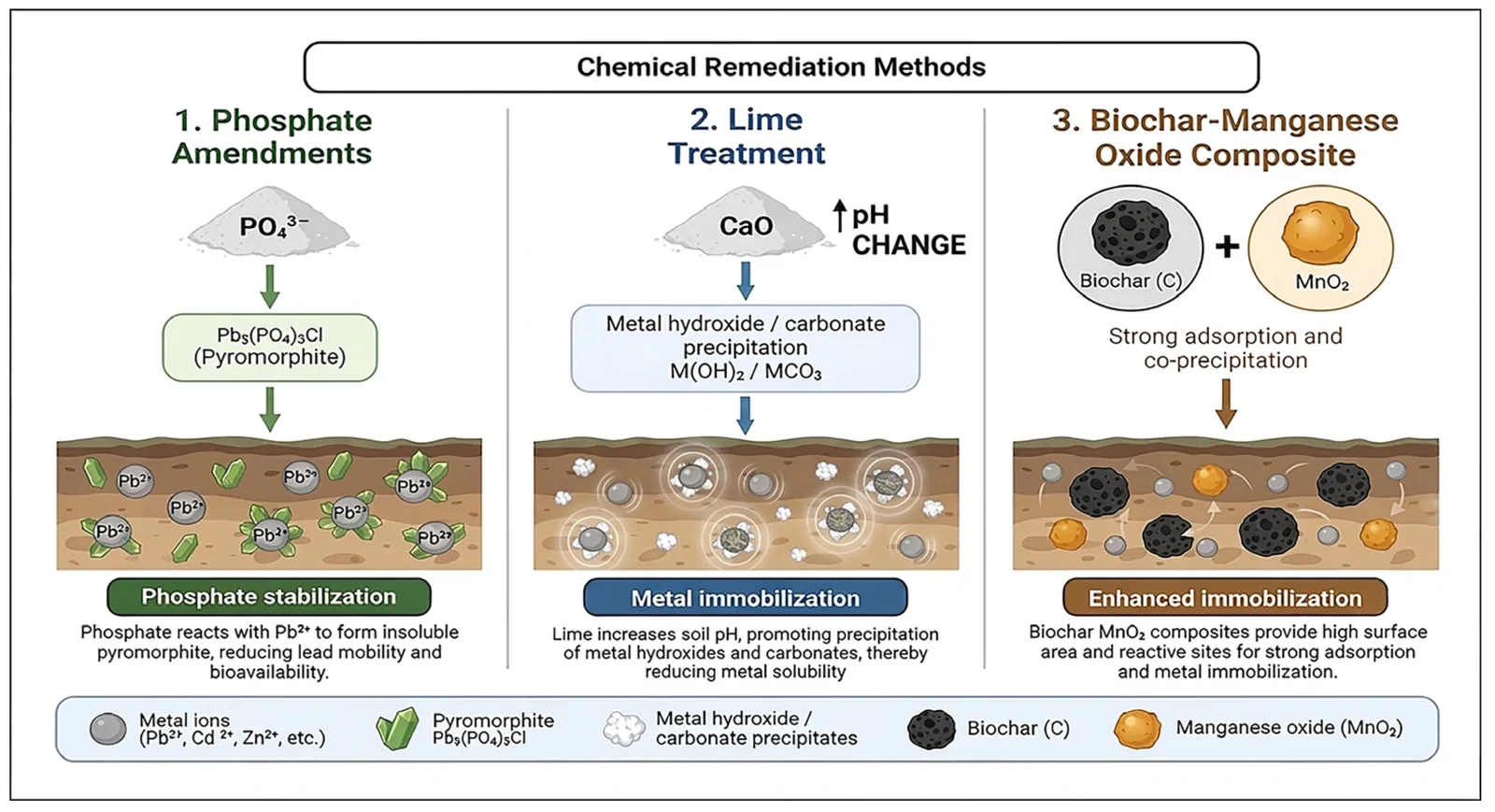
Schematic overview of chemical stabilization strategies for toxic metal remediation. Phosphate amendments convert Pb into insoluble pyromorphite; lime increases soil pH to induce hydroxide precipitation; and biochar/δ-MnO_2_ composites adsorb and immobilize metals [6,50]. The figure was generated with the assistance of ChatGPT image generation and subsequently modified using BioRender.

**Figure 4:**
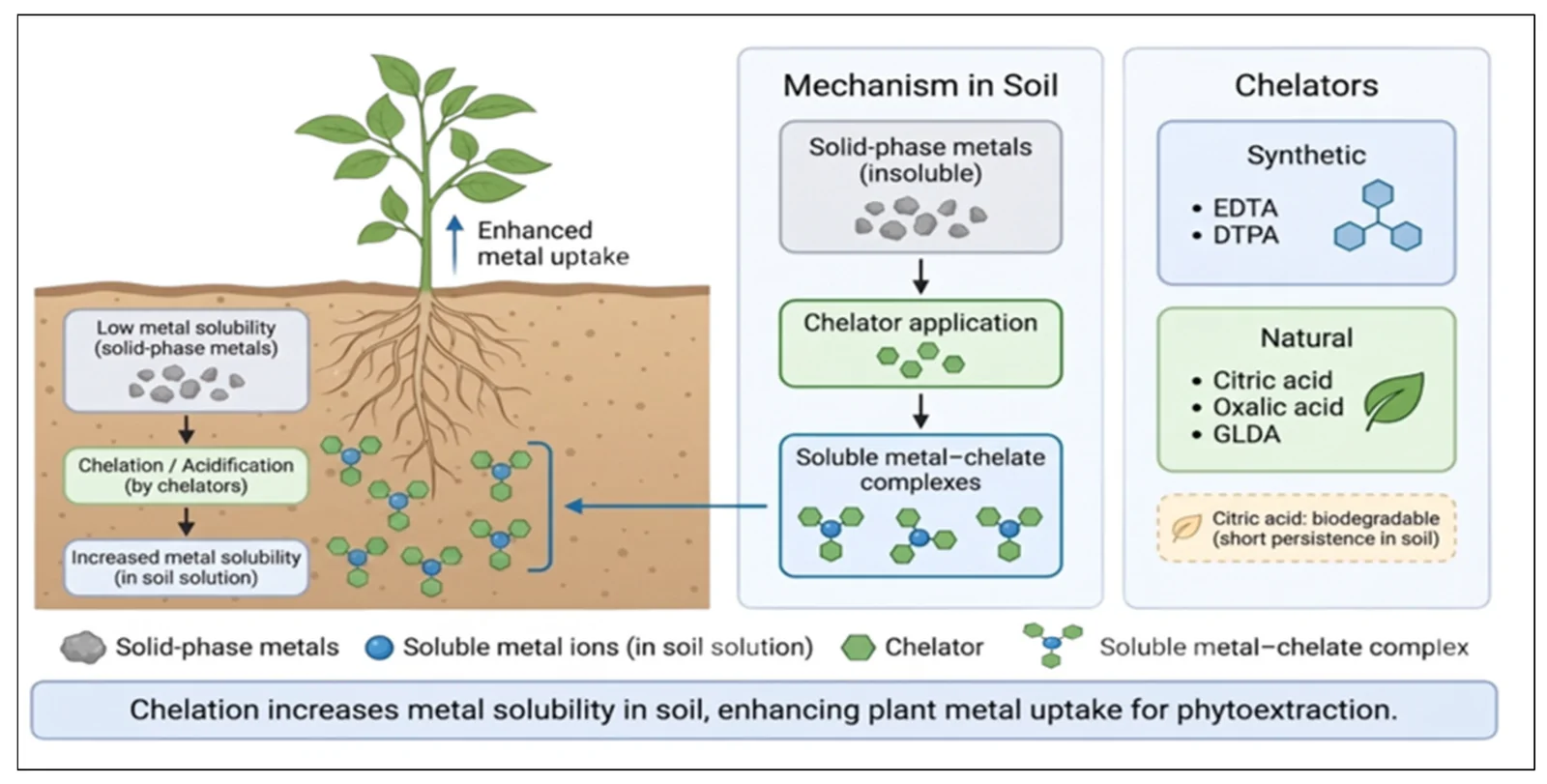
Illustration of chelation-assisted phytoextraction. Chelating agents (EDTA, DTPA, GLDA, citric acid, oxalic acid) increase metal solubility by desorbing metals from soil particles and forming soluble complexes, thereby enhancing plant uptake. The figure was generated with the assistance of ChatGPT image generation and subsequently modified using BioRender.

Chemical remediation constitutes a reaction-driven approach for managing toxic metal-contaminated soils by altering metal speciation, solubility, and mobility within the soil matrix. Unlike purely physical removal techniques, chemical methods primarily aim to immobilize or transform metals into less bioavailable forms, thereby reducing ecological and human health risks [6,50]. As illustrated in Figure 3 and Figure 4, the principal strategies include stabilization, immobilization, precipitation, adsorption, chelation-assisted extraction, and redox-mediated transformation.

### 7.1. Stabilization and Precipitation

Chemical stabilization reduces metal mobility by converting soluble or exchangeable metal fractions into sparingly soluble mineral phases. Phosphate amendments are among the most widely studied stabilizing agents, particularly for lead-contaminated soils. Phosphate reacts with Pb^2+^ to form pyromorphite [Pb_5_(PO_4_)_3_Cl], a highly stable and low-solubility mineral, significantly decreasing bioaccessible Pb fractions [12,13]. Similarly, lime-based treatments increase soil pH, promoting the precipitation of metal hydroxides and carbonates, thereby reducing the solubility of Cd^2+^, and Ni^2+^, [51]. Raising soil pH also enhances adsorption onto mineral surfaces and organic matter, further decreasing metal mobility [24]. While stabilization effectively reduces short-term bioavailability and leaching risk, it does not remove metals from the soil system. Long-term stability depends strongly on environmental conditions; fluctuations in pH, redox potential, or organic matter content may remobilize previously immobilized metals [24].

### 7.2. Adsorption and Ion Exchange Amendments

Adsorptive materials enhance metal immobilization through surface complexation, ion exchange, and co-precipitation mechanisms. Zeolite-based amendments possess crystalline structures with high cation exchange capacity, enabling efficient entrapment of metal ions such as Pb^2+^ and Cd^2+^ [52]. Biochar and biochar–manganese oxide composites further improve immobilization efficiency by providing large surface area, functional groups for complexation, and redox-active sites [31]. These materials not only reduce bioavailable metal fractions but may also improve soil structure and water retention. However, excessive amendment application may alter soil nutrient balance, microbial activity, and physical properties [9]. Additionally, immobilization strategies do not eliminate contamination, and long-term monitoring is required to ensure sustained stability.

### 7.3. Chelation-Assisted Phytoextraction

Chelation-assisted remediation represents an alternative chemical approach aimed at increasing metal solubility and facilitating plant uptake during phytoextraction (Figure 4). Chelating agents such as EDTA, DTPA, citric acid, oxalic acid, and GLDA form stable metal–ligand complexes, enhancing desorption from soil particles and promoting translocation into plant tissues [10,11]. This method can significantly improve metal uptake efficiency, particularly for Pb and Cd. However, synthetic chelators such as EDTA may increase metal mobility beyond the root zone, posing risks of groundwater contamination [10]. Furthermore, residual chelating agents may persist in the soil and disrupt the natural metal equilibrium. Biodegradable chelators offer a comparatively safer alternative, though their effectiveness may be lower under certain soil conditions [11]. The balance between enhanced extraction efficiency and environmental safety remains a central challenge.

### 7.4. Redox Transformation Mechanisms

Certain chemical amendments influence metal valence states through oxidation–reduction reactions, altering solubility and toxicity. For example, conversion of more mobile metal species into less soluble oxidation states can reduce environmental risk [50]. Conversely, redox fluctuations in contaminated soils may transform stable species into more toxic forms, emphasizing the importance of site-specific assessment. Redox-mediated stabilization is particularly relevant for metals such as chromium and arsenic, whose toxicity is strongly dependent on the oxidation state [42]. Effective redox management requires careful control of soil conditions to prevent unintended mobilization.

Although chemical remediation methods are generally faster than biological approaches and can achieve significant reductions in bioavailable fractions, several limitations constrain their long-term sustainability: secondary pollution risks: excess reagents, leachates, and by-products may require additional treatment, soil quality alteration where changes in pH and chemical composition may negatively affect soil fertility and microbial communities [9], cost and resource demand through repeated amendment applications increase operational costs, lack of metal removal where most chemical strategies immobilize rather than eliminate contaminants, and long-term stability uncertainty where environmental fluctuations may remobilize stabilized metals [24]. Consequently, while chemical remediation plays a critical role in risk reduction particularly in highly contaminated or industrial sites, its limitations underscore the need for integrative strategies that combine chemical amendments with phytoremediation and microbial mitigation to achieve sustainable soil restoration.

## 8. Phytoremediation: Plant-Based Strategies

Phytoremediation is a plant-based remediation strategy that utilizes the physiological, biochemical, and molecular capabilities of plants to remove, stabilize, or transform HMs into contaminated soils. As a nature-based approach, it offers a cost-effective, environmentally compatible alternative to conventional physical and chemical remediation methods [15,16]. Unlike excavation or chemical stabilization, phytoremediation aims not only to reduce contaminant levels but also to restore soil structure and ecological function. The efficiency of phytoremediation is influenced by plant species, metal type, soil physicochemical properties, and rhizosphere microbial interactions [16,53]. Soil pH, organic matter content, redox conditions, and cation exchange capacity directly affect metal bioavailability and plant uptake dynamics [38,39].

### 8.1. Phytoextraction

Phytoextraction involves the uptake of TMs from soil and their translocation to above-ground tissues, which are subsequently harvested to remove contaminants from the site. This process relies on metal transporters located in root cell membranes, chelation by phytochelatins and organic acids, and sequestration within vacuoles to minimize cytotoxicity [17,18]. Hyperaccumulator species such as *Brassica juncea*, *Thlaspi caerulescens*, and *Helianthus annuus* are capable of accumulating exceptionally high concentrations of Cd and Pb in shoot tissues [16]. However, a major limitation of hyperaccumulators is their relatively low biomass production, which restricts total metal removal capacity [18]. Consequently, achieving substantial remediation may require multiple cropping cycles.

### 8.2. Phytostabilization

Phytostabilization aims to reduce metal mobility and bioavailability in soils rather than remove contaminants. Plants immobilize metals within the rhizosphere through root adsorption, precipitation, and complexation with exudates such as organic acids and phenolic compounds [34]. Dense root systems enhance soil aggregation and reduce erosion, thereby limiting metal dispersion. Species such as *Vetiveria zizanioides* and *Festuca arundinacea* have demonstrated tolerance to elevated metal concentrations and are frequently used for stabilization of Pb- and As-contaminated soils [1,34]. While phytostabilization reduces immediate ecological risk, it does not eliminate contamination and requires long-term vegetation management.

### 8.3. Rhizofiltration

Rhizofiltration targets aqueous or waterlogged environments, where plant roots absorb, adsorb, or precipitate dissolved metals from contaminated water. Aquatic and semi-aquatic species such as *Eichhornia crassipes* and *Lemna minor* exhibit high metal uptake efficiency due to extensive root surface area and mucilage production [54]. This technique is particularly useful for treating irrigation water or drainage systems associated with contaminated soils. However, biomass disposal remains a critical consideration due to accumulated metal content.

### 8.4. Phytovolatilization

Phytovolatilization involves the uptake of specific elements followed by their transformation into volatile forms released into the atmosphere. This mechanism has been reported for elements such as selenium and mercury [54]. While it reduces soil concentrations, concerns remain regarding atmospheric redistribution and potential secondary exposure.

### 8.5. Phytodegradation and Indirect Effects

Although primarily relevant for organic contaminants, phytodegradation may indirectly influence toxic metal mobility through root exudation and enzymatic activity that alters soil chemistry [15]. Root exudates can modify the pH, enhance microbial activity, and influence metal solubility, thereby affecting overall remediation performance.

### 8.6. Plant–Microbe Interactions in Phytoremediation

**Figure 5:**
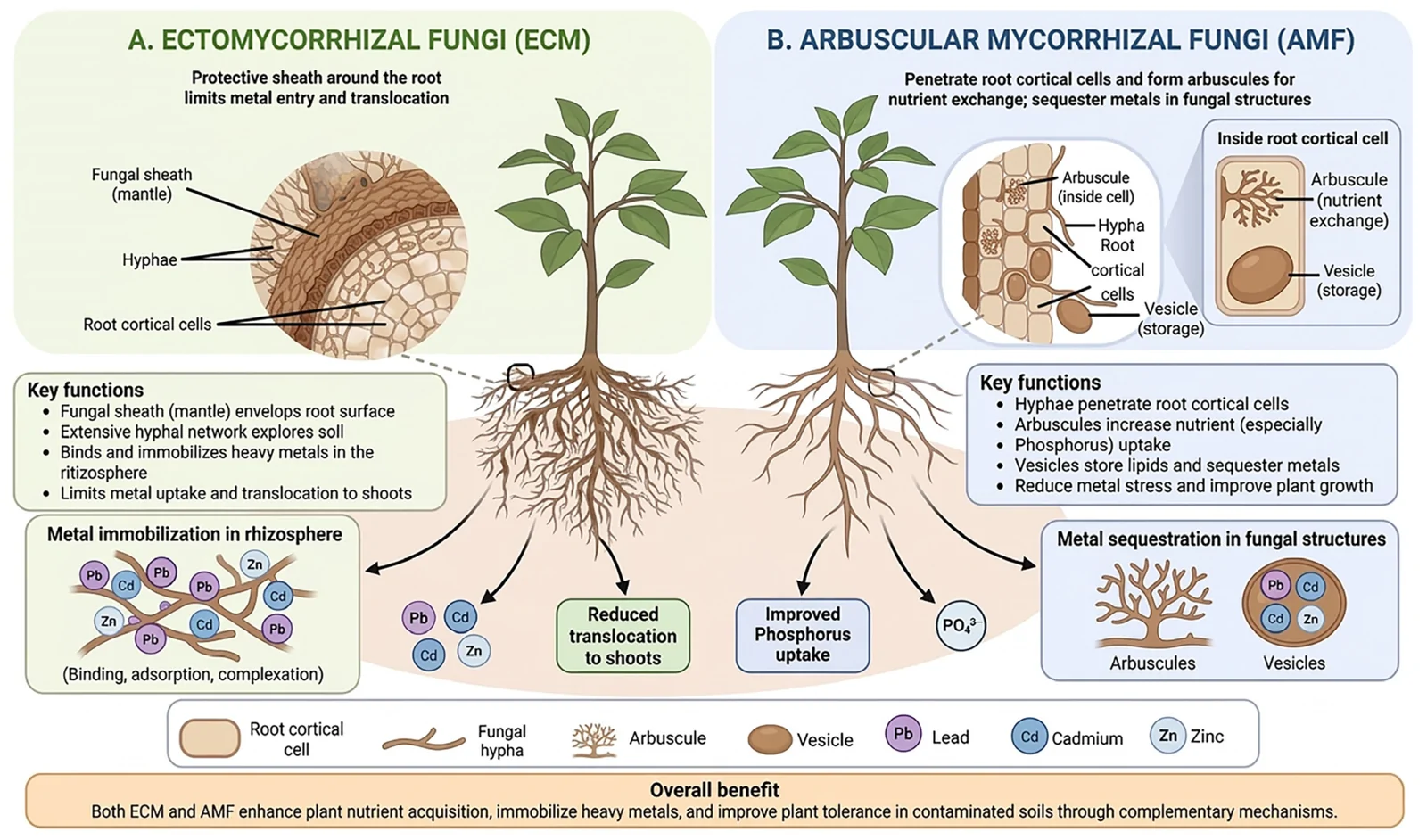
Comparison of ectomycorrhizal and arbuscular mycorrhizal fungi in plant nutrient and metal interactions. Arbuscular mycorrhizal fungi (AMF) colonize root cortical cells by forming arbuscules for nutrient exchange and vesicles for storage. Their extensive hyphal network in the rhizosphere increases phosphorus uptake and sequester HMs, reducing their bioavailability and protecting host plants under metal stress. Ectomycorrhizal fungi (ECM) colonize root surfaces by forming a dense fungal sheath (mantle) and an extensive hyphal network in the rhizosphere. This fungal structure binds and immobilizes TMs, limiting their entry into root cells and preventing transfer to shoots, thus enhancing host plant tolerance in contaminated soils [58]. The figure was generated with the assistance of ChatGPT image generation and subsequently modified using BioRender.

Plant growth-promoting rhizobacteria (PGPR) and mycorrhizal fungi play a critical role in enhancing phytoremediation efficiency. PGPR improves plant tolerance to toxic metal stress through mechanisms such as ACC deaminase activity, siderophore production, phosphate solubilization, and exopolysaccharide secretion [55,56]. These mechanisms reduce metal-induced ethylene production, enhance nutrient availability, and immobilize metals within the rhizosphere. Arbuscular mycorrhizal fungi (AMF), including *Glomus intraradices*, form symbiotic associations with plant roots, extending hyphal networks that enhance phosphorus uptake and sequester metals within fungal structures [57]. As illustrated in Figure 5, both AMF and ectomycorrhizal fungi contribute to metal immobilization and plant stress tolerance through distinct structural and physiological adaptations.

Despite its ecological advantages, phytoremediation faces several limitations like time requirement where remediation may require multiple growing seasons to achieve significant reductions, low biomass in hyperaccumulators which limits total metal extraction [18], metal bioavailability constraints: where aged or strongly bound metals may not be accessible for uptake [24], biomass disposal challenges where harvested tissues containing accumulated metals require safe management, and finally site-specific variability where soil heterogeneity affects performance consistency. Nevertheless, phytoremediation remains a promising strategy for large-scale agricultural soils due to its low cost, minimal environmental disturbance, and compatibility with ecosystem restoration goals [1,15].

## 9. Microbial Mitigation of TMs

Microorganisms play a central role in regulating toxic metal mobility, bioavailability, and toxicity in contaminated soils. Bacteria, fungi, and actinomycetes possess diverse physiological and biochemical mechanisms that enable them to tolerate, transform, immobilize, or detoxify metal contaminants [19,20]. Unlike purely chemical stabilization methods, microbial mitigation operates through dynamic biological processes that influence metal speciation and rhizosphere interactions. As summarized in Table 2, several microbial taxa demonstrate resistance to TMs through distinct mechanisms, including bioaccumulation, biosorption, biomineralization, and redox transformation.

### 9.1. Bioaccumulation

Bioaccumulation is an active, metabolism-dependent process in which living microbial cells transport metal ions into the cytoplasm and sequester them intracellularly. This mechanism typically occurs in two stages: initial passive adsorption onto the cell surface followed by energy-dependent transport across the cell membrane [21,63]. A species such as *Microbacterium* sp. has demonstrated the capacity to accumulate chromium (VI), while fungal strains such as *Fusarium oxysporum* can sequester iron and other metals within cellular compartments (Table 2). Intracellular sequestration often involves binding to metallothioneins or compartmentalization within vacuoles and reducing cytotoxic effects.

### 9.2. Biosorption

Biosorption is a passive physicochemical process independent of cellular metabolism. It involves adsorption of metal ions onto functional groups present on microbial cell walls, including carboxyl, hydroxyl, phosphate, and amino groups [63]. This process may proceed through ion exchange, surface complexation, or precipitation. Actinomycetes such as *Streptomyces* sp. BN3 and *Amycolatopsis* spp. Exhibit a strong biosorptive capacity for Pb, and Cr, as indicated in Table 2. Fungal species such as *Penicillium oxalicum* and *Fusarium solani* also demonstrate effective biosorption and surface-mediated immobilization of Cd [48]. Biosorption is particularly advantageous in soils with high metal concentrations because it rapidly reduces soluble fractions without requiring metabolic activity.

### 9.3. Biomineralization

Biomineralization refers to microbially mediated precipitation of stable mineral phases, such as metal carbonates, phosphates, or sulfides, effectively immobilizing metals within the soil matrix [19]. This process may occur intracellularly or extracellularly and contributes to long-term stabilization. For example, phosphate-solubilizing microorganisms can influence phosphorus cycling and indirectly promote precipitation of metal-phosphate minerals [64]. Fungal strains such as *Penicillium oxalicum* contribute to cadmium immobilization through organic-acid production and mineral formation [48]. Biomineralization is particularly important in reducing metal leachability and long-term environmental risk.

### 9.4. Redox Transformation

Microorganisms can alter metal valence states through enzymatically driven oxidation–reduction reactions, thereby modifying toxicity and mobility [20]. Redox transformations are especially relevant for metals such as chromium and arsenic, whose toxicity depends strongly on the oxidation state [42]. Bacterial species such as *Acidithiobacillus ferrooxidans* and *Shewanella putrefaciens* contribute to arsenic cycling by mediating redox-driven transformations that alter arsenic speciation, mineral associations, solubility and bioavailability (Table 2). These transformations may either detoxify metals or, under certain conditions, increase mobility, emphasizing the need for site-specific assessment.

### 9.5. Plant Growth-Promoting Rhizobacteria (PGPR)

Plant growth-promoting rhizobacteria (PGPR) enhance plant tolerance to toxic metal stress while contributing to metal immobilization or transformation. As summarized in Table 3, key PGPR mechanisms include: ACC Deaminase Activity: Reduces ethylene levels in plants under stress, promoting root growth and tolerance [55]; Siderophore Production: Chelates metal ions, influencing metal availability in the rhizosphere; Phosphate Solubilization: Enhances nutrient availability and indirectly supports plant resilience [64]; Exopolysaccharide production: Immobilizes metals through adsorption and ion exchange within the rhizosphere matrix (Table 3). Metal-tolerant bacterial strains have been shown to adjust superoxide dismutase, catalase, and glutathione-related antioxidant responses during metal exposure. These mechanisms collectively reduce metal-induced oxidative stress, enhance nutrient uptake, and improve plant biomass production in contaminated soils [20].

### 9.6. Mycorrhizal Associations

Arbuscular mycorrhizal fungi (AMF) and ectomycorrhizal fungi form symbiotic relationships with plant roots, extending hyphal networks that enhance nutrient acquisition and modulate metal uptake. AMF can sequester metals within fungal structures, limiting translocation to aerial plant parts [57]. As illustrated in Figure 5, these fungal networks contribute to both stabilization and plant tolerance. Mycorrhizal associations therefore serve as critical mediators in plant–microbe–metal interactions, enhancing phytoremediation performance under stress conditions.

### 9.7. Synthetic Microbial Consortia and Bioaugmentation

**Figure 6:**
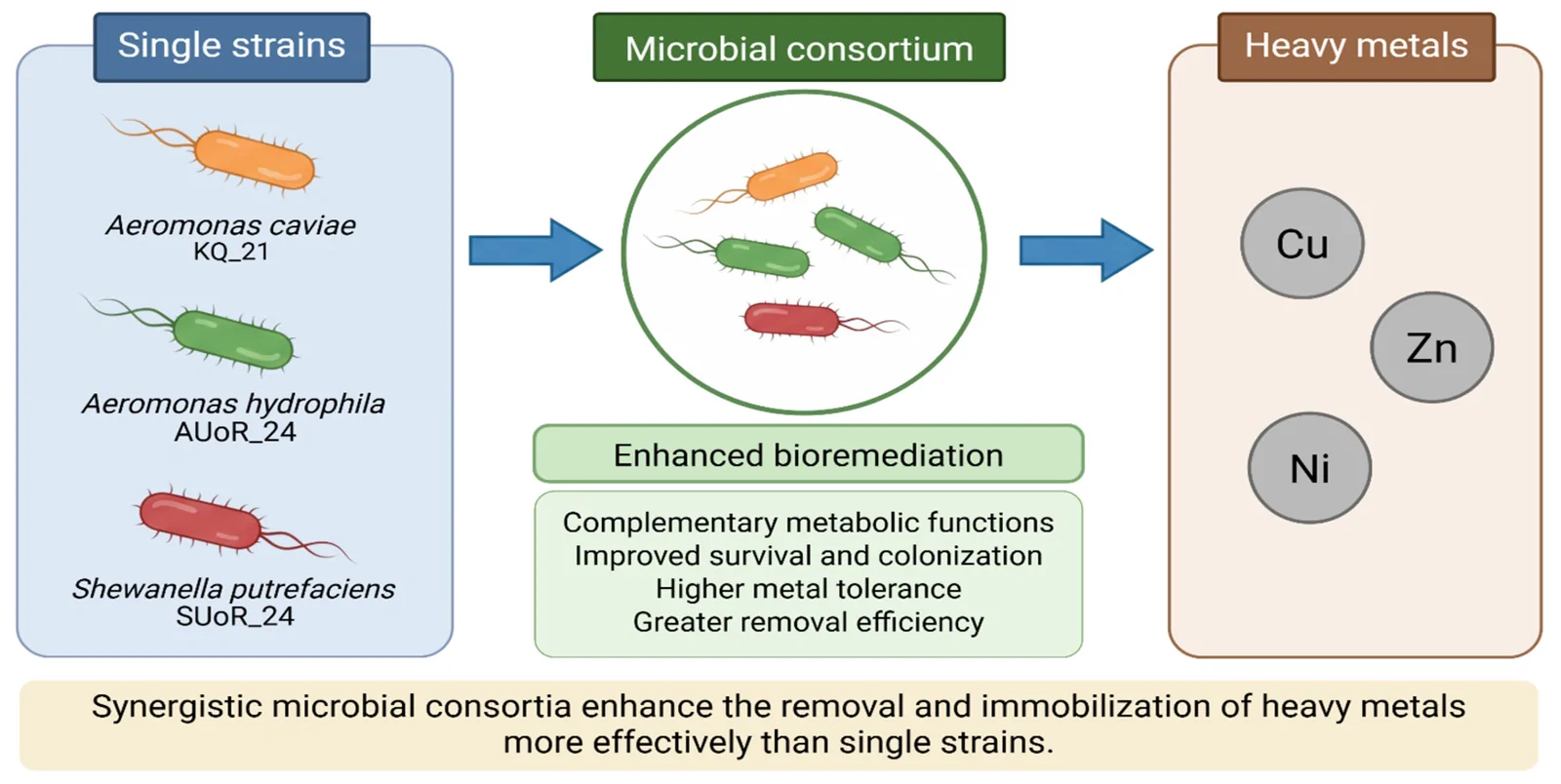
*Aeromonas caviae* KQ_21*, Aeromonas hydrophila* AUoR_24, and *Shewanella putrefaciens* SUoR_24 as an example of synthetic communities [20]. The figure was generated with the assistance of ChatGPT image generation and subsequently modified using BioRender.

Recent advances emphasize the use of engineered or synthetic microbial consortia to improve remediation efficiency. Mixed microbial communities integrating complementary metabolic traits such as biosorption, redox transformation, and phosphate solubilization often outperform single strains in multi-metal-contaminated soils [20]. Examples presented in the manuscript (Figure 6) include consortia containing *Aeromonas caviae*, *Aeromonas hydrophila*, and *Shewanella putrefaciens*, which demonstrated enhanced removal of Cu, Zn, and Ni compared to individual isolates. Bioaugmentation (introduction of specialized strains) and biostimulation (enhancing indigenous microbial activity) further support detoxification processes. However, microbial stability in field soils remains a limitation due to environmental variability and competition with native communities [20].

Although microbial remediation offers several advantages including low cost, ecological compatibility, and adaptability, it faces constraints: environmental sensitivity where temperature, pH, and moisture fluctuations affect microbial survival; competition with indigenous microbiota where introduced strains may fail to establish stable populations; incomplete metal removal where many mechanisms immobilize rather than eliminate metals; and site-specific variability where performance depends strongly on soil characteristics. Nevertheless, microbial mitigation represents a versatile and scalable approach, particularly when integrated with phytoremediation and soil amendments.

## 10. Integrated Plant–Microbe-Soil Engineering Framework for Sustainable Metal Remediation

Integrated phytoremediation combines plant-mediated metal extraction or stabilization with microbial regulation of metal speciation, nutrient availability, root development, and stress tolerance. As summarized in Table 4, plant- and microbe-based approaches exhibit complementary strengths and limitations: plants provide contaminant extraction, rhizosphere stabilization, and sustained soil cover, whereas microorganisms mediate metal transformation, nutrient cycling, and plant adaptation to metal stress. Their integration may therefore overcome important limitations of standalone treatments, including low biomass production, restricted metal bioavailability, and metal-induced phytotoxicity [1,20]. However, system performance depends on the compatibility among plant genotypes, microbial functional traits, contaminant properties, and site-specific environmental conditions. Integrated strategies should consequently be designed according to a predefined endpoint such as phytoextraction, phytostabilization, reduction in food-chain transfer, or restoration of soil functionality rather than on the assumption that all plant–microbe combinations are intrinsically synergistic.

### 10.1. Rhizosphere Synergy and Metal Bioavailability

The rhizosphere constitutes a biogeochemically active interface in which root exudates, microbial metabolites, and soil constituents interact to regulate metal mobility and biological availability. Root-derived organic acids, amino acids, and phenolic compounds modify rhizosphere pH, redox conditions, and metal complexation, thereby influencing contaminant solubility and uptake [34]. Plant growth-promoting rhizobacteria further modulate these processes through siderophore production, phosphate solubilization, ACC deaminase activity, and exopolysaccharide synthesis [55,64]. ACC deaminase reduces stress-induced ethylene accumulation and supports root development, whereas siderophores and extracellular polymers alter metal complexation and retention within the rhizosphere.

The outcome of these interactions depends on the intended remediation endpoint. Microbial mobilization may enhance phytoextraction by increasing the metal fraction accessible to plant roots, but excessive mobilization can increase leaching or phytotoxicity. Conversely, biosorption and biomineralization may support phytostabilization while restricting shoot translocation and total metal removal. Rhizosphere-assisted remediation should therefore be evaluated using metal-speciation analysis, root-to-shoot translocation, biomass-normalized extraction, and contaminant mass balance rather than tissue metal concentration alone.

### 10.2. Mycorrhizal Associations and Metal Sequestration

Arbuscular and ectomycorrhizal fungi extend the effective absorptive area of plant roots, improve nutrient and water acquisition, and modulate plant responses to metal stress [20,57]. Fungal structures can influence metal distribution through cell-wall adsorption, extracellular complexation, intracellular sequestration, and modification of rhizosphere chemistry. These processes may restrict metal translocation to aerial tissues and are therefore particularly relevant to phytostabilization and food-chain risk reduction.

Nevertheless, mycorrhizal effects vary with fungal species, host genotype, contaminant identity, exposure intensity, and soil nutrient status. Increased plant growth may also dilute tissue metal concentrations without reducing the total contaminant inventory. Accordingly, the performance of mycorrhizal-assisted remediation should be assessed through colonization stability, biomass production, metal distribution among plant organs, total metal removal or stabilization, and long-term changes in soil bioavailability.

### 10.3. Enhanced Phytoextraction Through PGPR Inoculation

**Figure 7:**
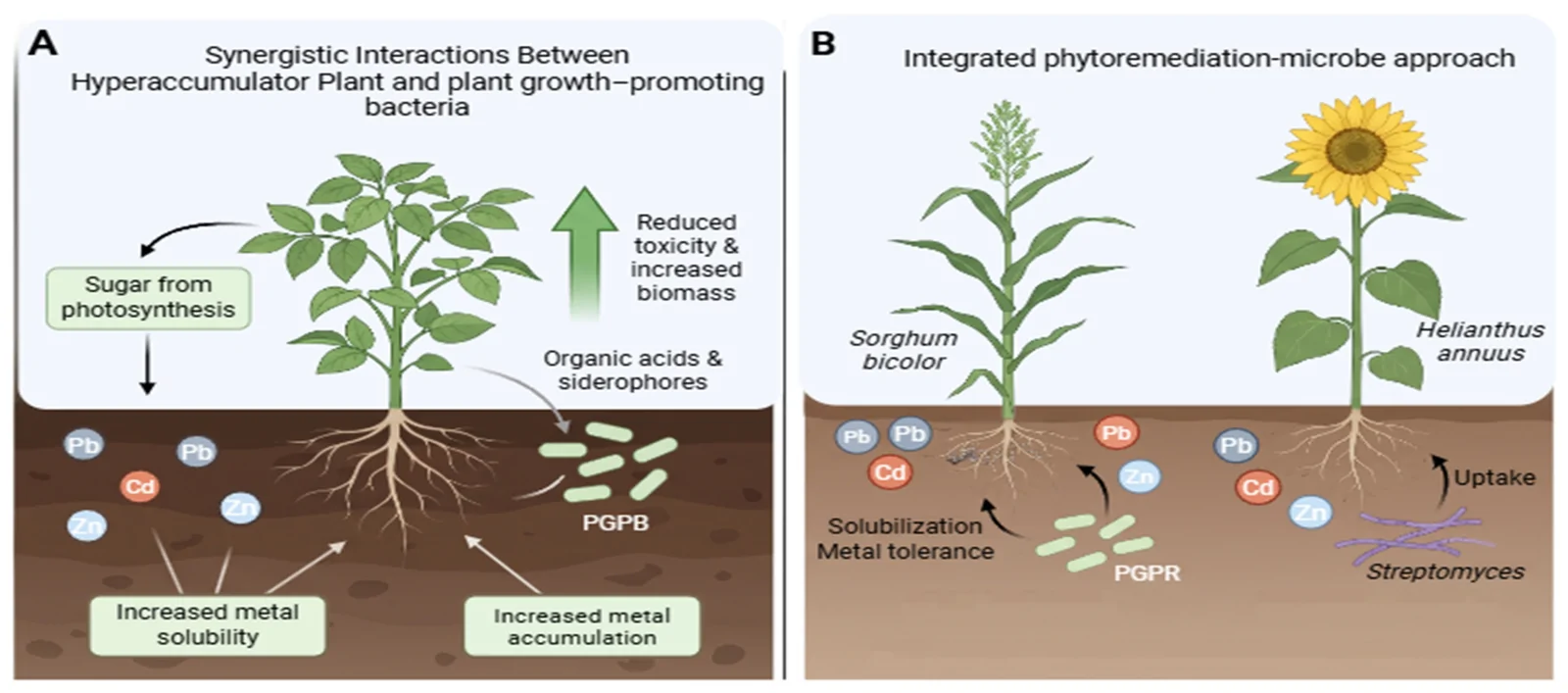
Plant–microbe synergy in phytoremediation. (**A**) Synergistic roles of plant growth-promoting bacteria in enhancing metal solubility and accumulation in hyperaccumulator plants and (**B**) Microbe-assisted phytoremediation: Enhancing metal uptake and tolerance in *Sorghum* and Sunflower. PGPR: Plant growth-promoting rhizobacteria; PGPB: Plant growth-promoting bacteria [20,77]. The figure was generated with the assistance of ChatGPT image generation and subsequently modified using BioRender.

Experimental and limited field evidence summarized in Table 4 indicates that PGPR inoculation can enhance plant biomass and metal accumulation under specific environmental conditions. These effects are mediated through stimulation of root development, improved nutrient acquisition, modification of rhizosphere metal availability, and regulation of plant stress-response pathways [20,77]. Associations between selected rhizosphere microorganisms and plants such as sorghum and sunflower may therefore improve plant establishment and metal tolerance while partially addressing the low biomass that frequently limit phytoextraction (Figure 7).

However, field performance remains dependent on plant–microbe compatibility, inoculum formulation, soil physicochemical properties, contaminant speciation, and the persistence of introduced strains. Competition with indigenous microorganisms and environmental fluctuations may substantially reduce inoculant survival and functionality. Furthermore, increased tissue metal concentrations do not necessarily indicate greater site-level removal, particularly when biomass production is limited. PGPR-assisted phytoextraction should therefore be evaluated using total metal removal per unit area, microbial persistence, biomass productivity, leaching potential, and performance across repeated harvesting cycles.

### 10.4. Microbial Consortia and Plant-Microbe Interactions in Bioremediation

Synthetic microbial consortia provide an opportunity to integrate complementary functions, including biosorption, biomineralization, redox transformation, nutrient solubilization, exopolysaccharide production, and plant-growth promotion. As shown in Figure 6, communities containing *Aeromonas caviae*, *Aeromonas hydrophila*, and *Shewanella putrefaciens*, for example, have been investigated for their capacity to mitigate multi-metal contamination through complementary metabolic activities [20]. Bioaugmentation introduces selected metal-tolerant microorganisms into contaminated soils, whereas biostimulation promotes the functional activity of indigenous microbial populations through targeted substrate or nutrient addition.

When combined with plants, these strategies may establish reciprocal interactions in which root-derived carbon supports microbial activity, while microorganisms regulate nutrient availability, metal transformation, and plant stress tolerance. Nevertheless, increased community complexity does not necessarily result in superior remediation. Competition, antagonism, functional redundancy, and environmental filtering may destabilize introduced consortia or eliminate critical functions following field application. Rational consortium design should therefore prioritize functional complementarity, host compatibility, ecological stability, and resilience across environmental conditions and successive plant-growth cycles.

### 10.5. Soil Health Restoration and Ecosystem Recovery

Integrated plant–microbe systems may contribute to soil recovery by improving aggregation, nutrient cycling, water retention, microbial diversity, and vegetation establishment [20]. As indicated in Table 4, these systems may also reduce contaminant accumulation in edible tissues and facilitate the restoration of degraded land. Unlike treatments that exclusively alter contaminant chemistry, biologically integrated systems can simultaneously influence contaminant risk and soil ecological function.

However, ecological recovery should not be inferred solely from increased plant biomass or decreased extractable metal concentrations. Immobilized metals may be remobilized following changes in pH, redox conditions, organic matter, or land management. Introduced microorganisms may also fail to persist because of competition, predation, or environmental stress. Moreover, substantial contaminant reduction may require multiple growing seasons, and metal-enriched biomass must be harvested and processed safely to prevent secondary contamination. Although integrated systems provide greater functional complementarity than standalone treatments, their long-term effectiveness and scalability remain site-dependent and require multi-season validation [1,20,26].

### 10.6. Conceptual Framework for Integrated-System Design and Translation

Integrated remediation can be conceptualized as a multilevel framework linking site-specific inputs, intervention components, biogeochemical processes, and remediation outcomes. Site characteristics—including contaminant identity, concentration, speciation, depth, soil pH, organic matter, hydrology, and intended land use define the primary design constraints. The intervention layer comprises selected plant genotypes, microbial inoculants or consortia, and, where scientifically justified, biochar or nano-enabled amendments. Interactions among these components regulate metal complexation, biosorption, biomineralization, redox transformation, nutrient acquisition, plant stress responses, and ultimately phytoextraction, phytostabilization, exposure reduction, and soil recovery [20,23,25].

These interactions may be synergistic, antagonistic, or functionally redundant. Sorptive amendments, for example, may reduce metal toxicity and promote plant establishment while simultaneously restricting the metal availability required for phytoextraction. Conversely, metal-mobilizing microorganisms may enhance plant uptake but also increase phytotoxicity or off-site transport. The system design must therefore align each component with the intended remediation endpoint and explicitly evaluate potential trade-offs.

Multi-omics approaches provide a mechanistic basis for component selection and system optimization. Metagenomics can identify microbial genes associated with metal resistance, transport, biomineralization, and nutrient cycling; transcriptomics and proteomics can determine whether these functions are expressed under contaminant stress; and metabolomics can characterize root exudates and microbial metabolites involved in metal transformation and plant–microbe communication [33]. Nevertheless, omics-derived associations require functional validation through metal-speciation analysis, contaminant mass balance, microbial-persistence measurements, plant performance, and indicators of ecological recovery.

Strategy selection should follow a site-specific, endpoint-oriented decision process. When contaminant removal is the objective and metals are accessible to plant roots, high-biomass or hyperaccumulating plants may be combined with microorganisms that moderately enhance metal availability. Where the priority is to restrict leaching, crop accumulation, or food-chain transfer, phytostabilizing vegetation should be combined with immobilizing microorganisms and sorptive amendments. Nano-enabled materials should be considered only when they provide a demonstrable advantage over conventional amendments and when their environmental fate and non-target ecotoxicity can be adequately characterized [28,29,30]. Climatic suitability, treatment duration, biomass management, regulatory acceptance, and life-cycle costs should also inform strategy selection.

Technology readiness should be evaluated for the complete remediation system rather than for individual components. Selected plant–microbe and biochar-assisted systems have progressed to pilot or field-demonstration stages, whereas synthetic consortia and nano-assisted systems remain predominantly at laboratory-to-pilot levels. Omics platforms are mature analytical technologies but remain less established as predictive tools for field-system design. CRISPR-engineered organisms intended for environmental release remain largely at the proof-of-concept stage because of unresolved biosafety, containment, and regulatory constraints [26,28,33,35]. Operational deployment should therefore follow a stage-gated pathway incorporating site characterization, laboratory treatability testing, greenhouse and pilot validation, replicated multi-season field trials, contaminant mass balance, ecological-risk assessment, life-cycle and techno-economic evaluation, regulatory authorization, and long-term monitoring. This framework shifts the evaluation of integrated remediation from isolated mechanistic responses toward demonstrable and durable reductions in contaminant exposure and ecological risk under field-relevant conditions.

## 11. Challenges, Risks, and Translational Barriers

Despite encouraging laboratory results, the translation of integrated phytoremediation and microbial mitigation to field-scale application remains constrained by inconsistent performance, ecological uncertainty, and limited regulatory and economic readiness. Contradictory findings often arise because metal speciation, contamination age, soil physicochemical properties, plant genotype, and microbial composition determine whether a treatment promotes metal extraction or immobilization. Microbial inoculants may increase metal availability through siderophore and organic-acid production but may alternatively reduce plant uptake through biosorption, biomineralization, and exopolysaccharide-mediated sequestration [20,23,25]. Similarly, biochar generally immobilizes metals through adsorption, precipitation, and pH modification, although its effectiveness varies with feedstock, pyrolysis conditions, application rate, and soil chemistry [31,32]. These context-dependent responses contribute to the frequent discrepancy between controlled experiments and field performance, where aged contaminants, soil heterogeneity, climatic variability, limited root depth, low plant biomass, and poor persistence of introduced microorganisms can substantially reduce remediation efficiency [24,26,77,78]. Nano-assisted remediation presents an additional benefit–risk trade-off: nanoparticles may enhance metal adsorption or uptake; yet, their persistence, dissolution, aggregation, and transformation can generate oxidative stress, release toxic ions, and disrupt non-target soil organisms [28,29,30,79]. A recent global meta-analysis incorporating 2,134 paired observations demonstrated that nanomaterials significantly reduced soil microbial biomass, activity, and function, with metal-based nanoparticles producing stronger adverse effects than carbon-based materials [80]. CRISPR/Cas engineering may enhance metal transport, chelator production, and stress tolerance; however, off-target mutations, unintended genomic rearrangements, genetic instability, gene flow, and horizontal transfer from engineered microorganisms remain important biosafety concerns [35,81,82]. Moreover, enhanced metal tolerance does not necessarily increase net remediation because reduced uptake may protect the organism without decreasing the soil contaminant inventory. Regulatory requirements for nano-enabled amendments and genome-edited organisms remain jurisdiction-dependent and may require environmental-release authorization, molecular characterization, containment, ecological-risk assessment, and post-release monitoring.

From an economic perspective, the feasibility of phytoremediation, microbial-assisted remediation, and integrated remediation strategies depends on a range of site-specific factors, including the extent and severity of contamination, treatment area and duration, site preparation, labor and monitoring requirements, land-use constraints, energy and material inputs, post-treatment management, and the handling or disposal of metal-enriched biomass. Economic feasibility is often overstated because long treatment periods, repeated cultivation, amendment or inoculum production, monitoring, land-use restrictions, and contaminated-biomass management are rarely incorporated comprehensively into experimental assessments [15,26,83]. Conventional physicochemical approaches may require substantial capital investment, energy consumption, specialized equipment, and waste-management infrastructure, whereas phytoremediation and microbial-based approaches generally involve lower physical disturbance and less energy-intensive infrastructure but may require prolonged maintenance, repeated cultivation, inoculation, or amendment application. Integrated plant–microbe systems may provide additional economic advantages by enhancing plant growth, improving metal immobilization or extraction efficiency, and potentially reducing remediation time; However, the production, formulation, storage, transport, and repeated application of microbial inoculants can introduce additional costs. Amendment-based systems may likewise incur recurring expenses associated with amendment production, transportation, application, and management of secondary waste streams. Consequently, comparisons between remediation strategies should consider whole-system and life-cycle costs rather than treatment costs alone. Phytomining may partially offset remediation costs through recovery of valuable metals from harvested biomass, but its economic viability depends on biomass productivity, tissue metal concentration, recovery efficiency, processing and energy requirements, and prevailing market conditions [83]. Technology readiness also remains uneven: selected phytostabilization and biochar-based systems have progressed to field demonstration, whereas nano-assisted remediation and synthetic microbial consortia remain largely at laboratory-to-pilot stages, and CRISPR-engineered organisms intended for environmental release are still predominantly at the proof-of-concept stage. Hence, remediation success should be evaluated not only in terms of enhanced metal uptake or immobilization but also through durable risk reduction, ecological safety, economic viability, scalability, and measurable restoration of soil functionality.

## 12. Conclusions

Successful restoration of toxic metal-contaminated soils cannot be reduced to maximizing metal uptake. Effective remediation requires alignment among contaminant speciation and bioavailability, soil physicochemical properties, the intended remediation endpoint, plant traits, microbial functions, amendment behavior, and post-harvest biomass management. Integrated systems offer their greatest advantage when their components perform complementary rather than antagonistic functions. This review advances a decision-oriented framework that connects these interacting factors with ecological safety, field deployability, economic feasibility, regulatory acceptance, and technology readiness. Future progress will require standardized, replicated, multi-season field trials that quantify contaminant mass balance, leaching and off-site transport, plant performance, microbial persistence, soil-function recovery, economic viability, and life-cycle impacts. Long-term monitoring, safe management of metal-enriched biomass, and rigorous assessment of the environmental fate and non-target effects of nano-enabled materials and engineered organisms will also be essential. Ultimately, translating mechanistic advances into safe, scalable, and sustainable field applications will depend on demonstrating durable reductions in contaminant exposure and ecological risk rather than short-term improvements in metal uptake or immobilization alone.

## Figures and Tables

**Table 1:** Representative global hotspots, dominant sources and biological implications of soil contamination by toxic metals.

Country	Priority Toxic Metals	Dominant Sources	Contamination Context	Representative Biological and Health Implications	References
China	Cd, Pb, As, Hg, Cr	Mining, smelting, industrial discharge, and fertilizer application	Widespread contamination of agricultural and industrial soils	Reduced seed germination and plant growth, oxidative stress, decreased microbial diversity, and increased neurological, renal, and carcinogenic risks	[24,38]
India	As, Cd, Cr, Pb	Agriculture activities, mining, and industrial effluents	Elevated concentrations in agricultural soils	Impaired photosynthesis and plant development, suppression of microbial activity and nitrogen cycling, and increased renal and carcinogenic risks	[39,40]
Spain and Poland	Pb	Historical mining and smelting	Legacy contamination in former mining; and smelting areas	Root-growth inhibition, chlorosis, reduced microbial biomass, and potential neurological and respiratory effects	[41,42]
Ghana/Nigeria	Hg	Artisanal and small-scale gold mining	Increasing contamination in mining districts	Reduced seed germination, plant growth inhibition, microbial toxicity, and neurological damage	[8]
Democratic Republic of Congo	Ni	Mining activities and naturally metal-rich ultramafic soils	Localized anthropogenic and geogenic enrichment near mining areas	Chlorosis, growth inhibition, disruption of microbial nutrient cycling, dermatitis, and potential systemic toxicity	[43]
Saudi Arabia	Pb, Ni, Cr	Oil production, industrial activities and urbanization	Localized enrichment in urban, industrial and agricultural soils	Reduced root and shoot growth, oxidative stress, decreased microbial diversity, and respiratory or dermal risks	[40,43]
United Arab Emirates	Pb, Ni	Urbanization, industry, and traffic emissions	Elevated concentrations in urban and industrial soils	Chlorosis, stunted plant growth, altered soil enzyme activity, and potential neurological effects	[40]
Qatar	Pb, Ni	Industrial activities, traffic and construction	Localized contamination in rapidly developing urban and industrial areas	Reduced seed germination and plant growth, suppression of microbial activity, and potential renal and hepatic effects	[43]
United States	Cd, Pb	Fertilizer application and industrial activities	Accumulation in agricultural and industrially affected soils	Plant growth inhibition, altered microbial enzyme activity, and increased renal and skeletal risks	[39]
Peru and Chile	Cd, Pb, As	Mining, smelting and geogenic inputs	High contamination near mining and smelting districts	Impaired photosynthesis, oxidative stress, reduced microbial diversity, and increased carcinogenic and organ-toxicity risks	[42]
Mexico	Hg	Illegal and artisanal gold mining	Increasing contamination and environmental risk in mining areas	Root-growth inhibition, chlorosis, microbial toxicity, and neurological damage	[8]

**Table 2:** Examples of microorganisms involved in toxic metal remediation and their associated mechanisms.

Microorganism	Microbial Group	Target Metal	Mechanism
*Acidithiobacillus ferrooxidans* and *Shewanella putrefaciens*	Bacteria	Arsenic	Enzymatic redox transformation [59]
*Microbacterium* sp.	Bacterium	Chromium VI	Bioaccumulation [59]
*Streptomyces* sp. BN3	Actinomycete	Lead	Cell-wall biosorption [60]
*Amycolatopsis* spp.	Actinomycete	Lead and Chromium	Surface complexation/ion-exchange [60]
*Fusarium oxysporum*	Fungus	Iron	Bioaccumulation [61]
*Fusarium solani* and *Penicillium oxalicum*	Fungi	Cadmium	Biosorption/biomineralization [62]

**Table 3:** Microbial-mediated mechanisms contributing to toxic metal mitigation and enhanced plant tolerance under metal stress.

Mechanism	Process and Representative Microorganisms	Effect on Plants Under Toxic Metal Stress	References
ACC deaminase	Degradation of plant-released ACC into ammonia and α-ketobutyrate. Reported in *Pseudomonas*, *Bacillus*, *Enterobacter* and *Azospirillum* spp.	Reduces intracellular ethylene and sustains plant growth under stress	[65]
Siderophore production	Chelation of toxic metals and sequestration. Common in *Pseudomonas*, *Bacillus* and *Streptomyces*.	Lowers free metal ion pools, alleviates oxidative stress, and enhances biomass	[66]
Phosphate solubilization	Secretion of organic acids (e.g., gluconic acid) that dissolve insoluble phosphate. Found in *Bacillus* and *Pseudomonas*.	Increases plant-available P, improves nutrition and growth under metal stress	[67]
Exopolysaccharide formation	Production of exopolysaccharides with high adsorption and ion-exchange capacity. Produced by *Bacillus*, *Pseudomonas* and other rhizobacteria.	Immobilizes TMs in the rhizosphere, reduces bioavailability to plants	[68]
Superoxide dismutase (SOD)	Microbial and plant-associated antioxidant enzymes convert superoxide radicals into hydrogen peroxide.	First-line ROS defense	[20]
Catalase	Converts hydrogen peroxide to water and oxygen.	Protects plant cells from oxidative injury	[69]
Peroxidases	Detoxify hydrogen peroxide using electron donors.	Limits oxidative membrane damage	[20]
Glutathione system	Redox buffering and metal complexation.	Supports detoxification and metal sequestration	[70]
Thioredoxin system	Maintains protein thiol status.	Protect enzymes under oxidative stress	[71]

**Table 4:** Comparative assessment of phytoremediation, microbial-assisted, and integrated biological strategies for toxic metal-contaminated soil restoration.

Strategy	Main Mechanism	Advantages	Limitations and Risks	References
Phytoextraction	Root uptake, intracellular chelation, vacuolar sequestration, xylem loading, and translocation of contaminants to harvestable shoots	Removes contaminants from soil; minimally disruptive; potentially compatible with biomass valorization and metal recovery	Slow treatment; limited by contaminant bioavailability, rooting depth, plant biomass, and phytotoxicity; contaminated biomass requires safe management	[2]
Phytostabilization and phytomanagement	Root sorption, precipitation, rhizosphere immobilization, erosion control, and reduction in contaminant transfer to crops or groundwater	Rapidly reduces contaminant mobility and exposure; improves soil cover, structure, and ecological function	Does not remove contaminants; effectiveness may decline with changes in soil pH, redox conditions, vegetation, or land use; requires long-term monitoring	[72]
PGPR/PGPB-assisted phytoremediation	ACC deaminase activity, phytohormone production, nutrient solubilization, siderophore production, exopolysaccharide formation, antioxidant support, and modification of contaminant bioavailability	Enhances plant biomass, root growth, stress tolerance, nutrient acquisition, and either phytoextraction or phytostabilization, depending on microbial traits	Inoculants may fail to colonize or persist; outcomes are strain-, plant-, contaminant-, and soil-specific; mobility may increase leaching risk	[2]
Mycorrhizal- and endophyte-assisted phytoremediation	Extension of the effective root system, nutrient acquisition, fungal sequestration, cell-wall binding, modulation of root-to-shoot transfer, and enhancement of plant antioxidant defense	Improves plant establishment and survival in nutrient-poor or contaminated soils; may reduce phytotoxicity and strengthen rhizosphere function	Strong host specificity; variable colonization; fungal sequestration may reduce shoot accumulation and therefore conflict with phytoextraction objectives	[73]
Microbial remediation using individual strains or indigenous communities	Biosorption, bioaccumulation, efflux, biomineralization, bioleaching, redox transformation, and enzymatic detoxification	Low soil; disturbance mechanical versatility; it can transform contaminant speciation and support recovery of soil biological functions	Often immobilizes or transforms rather than removes contaminants; activity is sensitive to pH, temperature, moisture, nutrients, and competition with native microorganisms	[74]
Synthetic microbial consortia	Functional complementarity among strains performing biosorption, redox transformation, nutrient mobility, biofilm formation, and plant-growth promotion	May provide broader contaminant tolerance, metabolic redundancy, and greater stability than single-strain inoculation	Community composition may change after application; antagonism, competition, biosafety, and reproduction remain concerns; difficult to manufacture and regulate consistently	[20]
Chelator-assisted plant–microbe systems	Chemical or microbial ligand production increases contaminant solubility and root accessibility; PGPR subsequently promotes plant growth and uptake	Can improve extraction of poorly available contaminants and increase root-to-shoot transport	Excessive mobility may cause plant toxicity, nutrient imbalance, movement below the root zone, and groundwater contamination; persistent synthetic chelators are a major concern	[75]
Omics-guided and engineered biological systems	Metagenomic and transcriptomic identification of functional traits; targeted modification of transporters, metal-binding proteins, antioxidant pathways, efflux systems, and detoxification genes	Enables rational selection or engineering of plants and microorganisms with complementary remediation functions	Limited field evidence; genetic instability, off-target effects, ecological release, gene transfer, regulatory barriers, and public acceptance must be addressed	[76]

## Data Availability

No new data were created or analyzed in this study. Data sharing is not applicable to this article.
